# Survival Comparison of Neoadjuvant Chemotherapy Followed by Irreversible Electroporation Versus Conversional Resection for Locally Advanced Pancreatic Cancer

**DOI:** 10.3389/fonc.2020.622318

**Published:** 2021-02-02

**Authors:** Chaobin He, Shuxin Sun, Xin Huang, Yu Zhang, Xiaojun Lin, Shengping Li

**Affiliations:** ^1^ Department of Pancreatobiliary Surgery, State Key Laboratory of Oncology in South China, Collaborative Innovation Center for Cancer Medicine, Sun Yat-sen University Cancer Center, Guangzhou, China; ^2^ State Key Laboratory of Ophthalmology, Zhongshan Ophthalmic Center, Sun Yat-sen University, Guangzhou, China

**Keywords:** locally advanced pancreatic cancer, conversional resection, irreversible electroporation, chemotherapy, prognosis

## Abstract

Locally advanced pancreatic cancer (LAPC) is a lethal disease and neoadjuvant chemotherapy and conversional resection is shown to provide the best survival for LAPC patients. Irreversible electroporation (IRE) is a new and effective method for the treatment of LAPC. This study aimed to compare the long-term survival of LAPC patients after neoadjuvant chemotherapy followed by conversional resection and IRE. A total of 140 LAPC patients were included from August 2015 to March 2020. The survival outcomes of patients after treatment with chemotherapy, chemotherapy combined with conversional resection or IRE were analyzed and compared. Patients in these three groups had similar clinical and pathological characteristics. Patients in the resection and IRE groups had similar median OS time (resection group vs. IRE group: 25.3 months vs. 26.0 months, P>0.050), which was significantly longer than that of the chemotherapy group (8.7 months, P<0.001). Additionally, patients in the resection and IRE groups had a median PFS of 10.6 and 12.0 months, respectively. Also, they were significantly higher than that of patients in the chemotherapy group. Chemotherapy combined with conversional resection and IRE was identified as significant prognostic factors for OS and PFS in LAPC patients. It was shown that compared with neoadjuvant chemotherapy followed by surgical resection, chemotherapy and IRE provided similar OS and PFS for LAPC patients with minimal invasion. This combination therapy may be a suitable treatment for LAPC patients.

## Introduction

Pancreatic cancer is the most lethal gastrointestinal cancer. Surgical resection, which represents the only path to cure, is suitable for only 20% of all patients (1). As a major part of this disease, locally advanced pancreatic cancer (LAPC) has been classified as unresectable cases with conventional surgical techniques. The optimal treatment for LAPC remains an open research question, given the lack of prospective studies of the latest chemotherapy regimens. Although the development of chemotherapy provided survival benefit for LAPC, the survival of LAPC after chemotherapy was also unsatisfied (2). Additional studies are underway to further delineate the chemotherapy that provides more survival benefits.

As the only curative option for pancreatic cancer, complete surgical remains an optional method for LAPC after neoadjuvant chemotherapy. The conversional surgery could provide more survival benefits than chemotherapy alone for these patients. A systemic review of studies investigating FOLFIRINOX (leucovorin, fluorouracil, irinotecan, and oxalipatin) as first-line treatment for LAPC revealed an average resection rate of 28% (0%–43%) across 12 studies (3), which indicated that the modified chemotherapy regimens might provide more chances of surgical resection for LAPC. Even after neoadjuvant chemotherapy, the curative resection of tumor remained a complex procedure with relatively high complication rates (4), which may partly prevent patients from obtaining much survival benefit. Moreover, large proportions of cases still have no chances to receive radical resection even after neoadjuvant chemotherapy. This unmet need has prompted researchers and practitioners to examine novel treatments and to optimize common therapeutic approaches. Considering the increasing control rates of primary tumor, most cases with LAPC were unaltered other than remission in terms of vascular infiltration. Local destructive therapies are worthy of being considered and tried with varying degrees of success.

Irreversible electroporation (IRE) is a novel non-thermal ablative method and it is often used for certain solid tumors that are unsuitable for surgery or thermal ablation due to the precarious anatomic location (5). More and more studies had shown that IRE acted as a useful method for the treatment of LAPC (6–9). Although compared with chemotherapy alone, better survival of LAPC patients after the combination therapy of IRE and chemotherapy was observed (9), the current experience of the comparisons of IRE and conversional resection after neoadjuvant chemotherapy in terms of survival elevation in PDAC patients is limited. The minimally invasive nature makes IRE attractive as the substitutable method of conversional resection. In this study, we aimed to compare the survival of LAPC patients after IRE combined with chemotherapy and conversional resection.

## Materials and Patients

### Patient Selection

Primary LAPC patients who were initially treated with chemotherapy from August 2015 to March 2020 were retrospectively analyzed in this study. All patients were pathologically confirmed pancreatic adenocarcinoma and radiologically confirmed LAPC. LAPC was defined per the National Comprehensive.

Cancer Network (NCCN) for pancreatic cancer, which describes LAPC as arterial encasement of either the celiac axis or superior mesenteric artery or unreconstructable superior mesenteric or portal vein involvement, with no evidence of metastatic disease from abdominal and thoracic computed tomography (CT) (10). After initial assessment and review by pancreatic tumor multidisciplinary team (MDT), these patients were confirmed to have LAPC and not borderline resectable tumors. Usually, patients would receive 4 months of chemotherapy. After the neoadjuvant chemotherapy, patients received radical resection, IRE or chemotherapy according to the assessment of tumor status made by the pancreatic tumor MDT. Patients whose tumors that were converted to resectable ones after neoadjuvant chemotherapy had received radical resection (R0 resection) and those whose tumors remained unresectable received IRE treatment or chemotherapy. Also, there was a proportion of patients had received IRE directly after the diagnosis of LAPC. All patients had received adjuvant chemotherapy after IRE or surgical resection. The exclusion criteria were as follows: (1) detected distant metastases; (2) an Eastern Cooperation Oncology Group performance status (ECOG PS) score larger than 2; and (3) missing or incomplete follow-up information. This study was approved by the Institutional Review Board of Sun Yat-sen University Cancer Center. All procedures involving human participants were in accordance with the ethical standards of the 1964 Helsinki Declaration and its later amendments.

### Data Collection

Several clinical and pathological data were retrospectively collected from medical records archived at Sun Yat-sen University Cancer Center, including age, gender, tumor size, tumor grade, tumor site, white blood cell (WBC) count, platelet (PLT) count, serum levels of alanine transaminase (ALT), aspartate aminotransferase (AST), alkaline phosphatase (ALP), glutamyl transpeptidase (GGT), albumin (ALB), total bilirubin (TBIL), indirect bilirubin (IBIL), C-reactive protein (CRP), and hepatitis B surface antigen (HBsAg), carcinoembryonic antigen (CEA), carbohydrate antigen 19-9 (CA19-9), and chemotherapy regimens. Overall survival (OS) and progression-free survival (PFS), which were defined as the duration from the date of diagnosis to death from all causes and tumor progression, respectively, or last follow-up, were endpoints of this study. Follow-up date ended at August 30, 2020.

### Treatment Procedure

Induction chemotherapy of either FOLFIRINOX or Abraxane-GEM (AG) was used for all included patients for four months (totaling three cycles of AG or 4–6 cycles of FOLFIRINOX-based chemotherapy). In terms of adjuvant chemotherapy, FOLFIRINOX, AG and Tegafur, Gimeracil, and Oteracil Porassium Capsules (S-1) were performed for these patients, which was in accordance with previous studies (9). Standard procedure of Whipple or distal pancreas resection with or without vascular resection was conducted for patients who had received radical resection. Uniform procedure of IRE which was reported in our previous studies (8) was adopted in the present study. Two to six probes would be used according to the tumor size and location to create an electric field around the tumor. The generator unit software is used to analysis the probe configuration data of ultrasound and provides the optimal voltage and pulse length delivery. An electric field of 1,500 V/cm, a pulse length of 70–90 ms, and a total of 90 pulses were used as the initial setting.

### Follow-Up

Regular follow-up was conducted for each included patient: 1 month after IRE or resection for the initial follow-up, and every 2–3 months thereafter. Abdominal CT or MRI, physical examination, and serum CA19-9, CEA tests were performed for each follow-up.

### Statistical Analysis

The chi-square test and Fisher’s exact test were used to compare categorical data, which are shown as frequencies and proportions. Variables that were significantly associated with OS were analyzed in the multivariate analysis using the Cox regression model to determine the independent predictive factors, along with the corresponding 95% confidence interval (CI). Survival differences were compared with log-rank test. All statistical analyses were performed using R version 3.4.2 software (The R Foundation for Statistical Computing, Vienna, Austria. http://www.r-project.org). A two tailed P-value was considered statistically significant if < 0.05.

## Results

### Patient Characteristics

**Figure 1:**
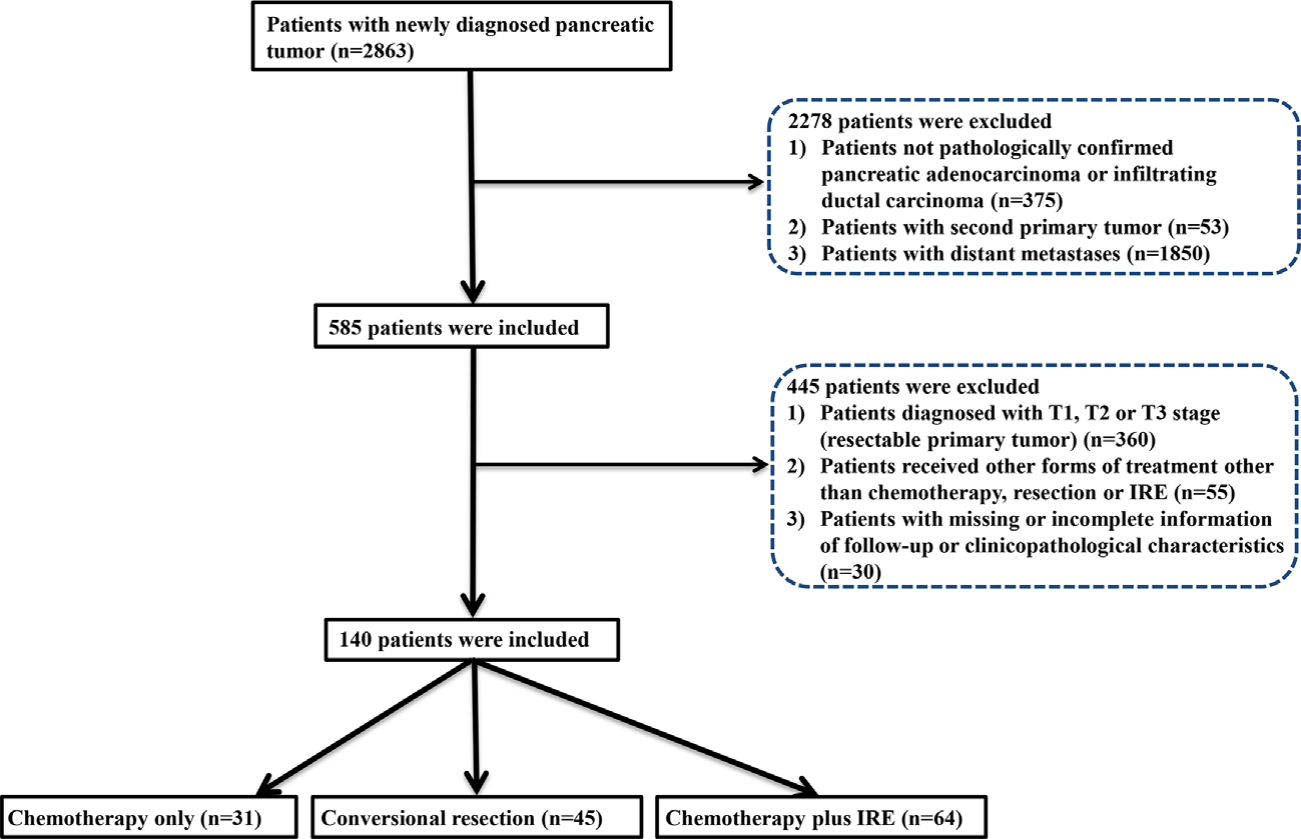
Flowchart of the included patients.

The flow diagram for data selection was shown in **Figure 1**. In total, 140 patients was included into this study, including 31 patients who received chemotherapy (chemotherapy group), 45 patients who received neoadjuvant chemotherapy and conversional resection (resection group), and 64 patients who received combination therapy of chemotherapy and IRE (IRE group). In the IRE group, there were 44 patients who received neoadjuvant chemotherapy while the remaining 20 patients did not receive neoadjuvant chemotherapy. The adjuvant chemotherapy was used for all patients in the resection and IRE groups. The baseline clinical and pathological characteristics were compared among three groups (**Table 1**). The median age was 60 years (range, 39–80 years), 59 years (range, 39–70 years), and 59 years (range, 34–87 years) for patients in the chemotherapy, resection, and IRE groups, respectively. Most patients were female patients in both chemotherapy and resection groups while the male patients were a little more than female patients in the IRE group. Most tumors were located in the head of pancreas among three groups. Compared with tumors in the chemotherapy group, those in the resection and IRE groups were more likely to be smaller than 4 cm. The levels of tumor markers, including CA19-9 and CEA, were similar among three groups. Additionally, patients in all three groups had comparable chemotherapy regimens.

**Table 1:** Clinicopathological characteristics of patients with PDAC stratified by tumor site.

ContinuousVariables*	Treatment				Category variables	Treatment				
	Chemotherapy	Conversionalsurgery	Chemotherapy + IRE	P		Chemotherapy	Conversionalsurgery	Chemotherapy + IRE	N	P
Age (years)	60.23 (56.02-64.40)	53.81 (38.84–68.76)	58.36 (55.86–60.86)	0.412	Gender					0.149
Tumor size (cm)	4.66 (3.85–5.47)	3.83 (3.32–4.34)	3.95 (3.60–4.30)	0.105	Male	12 (38.7%)	16 (35.6%)	34 (53.1%)	62	
WBC (*10^9^)	6.76 (6.05–7.48)	6.89 (6.09–7.69)	5.93 (5.42–6.44)	0.061	Female	19 (61.3%)	29 (64.4%)	30 (46.9%)	78	
HGB (g/L)	125.32 (120.67–129.97)	123.21 (117.06–129.37)	128.88 (125.06–132.69)	0.207	Adjuvant chemotherapy type					0.336
PLT (*10^9^)	224.35 (193.64–255.06)	231.14 (202.69–259.59)	240.32 (208.44–243.84)	0.610	S-1	11 (35.5%)	12 (26.7%)	25 (39.1%)	48	
ALT (U/L)	45.97 (24.51–67.44)	57.35 (37.02–77.68)	63.48 (37.69–89.26)	0.512	AG	5 (16.1%)	11 (24.4%)	18 (28.1%)	34	
AST (U/L)	27.54 (19.81–35.26)	39.53 (22.60–56.46)	47.65 (32.74–62.55)	0.423	FOLFIRINOX	15 (48.4%)	22 (48.9%)	21 (32.8%)	58	
ALP (U/L)	136.06 (75.72–196.40)	152.92 (133.57–172.27)	146.55 (109.53–183.57)	0.474	Tumor grade					0.666
GGT (U/L)	112.29 (14.19–210.39)	159.07 (126.88–191.26)	178.51 (93.46–263.56)	0.210	Well/Moderate	13 (41.9%)	21 (46.7%)	33 (51.6%)	67	
ALB (g/L)	42.01 (40.89–43.13)	40.03 (38.38–41.69)	43.62 (42.81–44.42)	0.111	Poor	18 (58.1%)	24 (53.3%)	31 (48.4%)	73	
TBIL (umol/L)	20.13 (9.67–30.56)	35.44 (21.37–49.51)	43.29 (22.92–63.67)	0.217	Tumor site					0.330
IBIL (umol/L)	16.51 (14.16–18.86)	19.82 (13.17–26.47)	17.18 (15.50–18.86)	0.662	Head	19 (61.3%)	26 (57.8%)	30 (46.9%)	75	
CRP (ng/L)	7.23 (2.15–12.32)	15.93 (7.05–24.81)	7.33 (2.78–11.88)	0.095	Body/Tail	12 (38.7%)	19 (42.2%)	34 (53.1%)	65	
CEA (ng/ml)	57.10 (1.22–137.48)	9.22 (3.80–14.63)	19.20 (3.29–35.11)	0.159						
CA19-9 (U/ml)	2,077.04 (204.71–3,949.37)	714.43 (242.82–1,186.03)	1,043.54 (350.49–1,736.60)	0.169						

WBC, white blood cell; PLT, platelet; ALT, alanine transaminase; AST, aspartate aminotransferase; ALP, alkaline phosphatase; GGT, glutamyl transpeptidase; ALB, albumin; TBIL, total bilirubin; IBIL, indirect bilirubin; CRP, C-reactive protein; HBsAg, hepatitis B surface antigen; CEA, carcinoembryonic antigen; CA19-9, carbohydrate antigen 19-9; AG, Abraxane-GEM; FOLFIRINOX, leucovorin, fluorouracil, irinotecan, and oxalipatin. * Median value and 95% confidence interval.

### Survival Comparisons Among Three Groups

**Figure 2:**
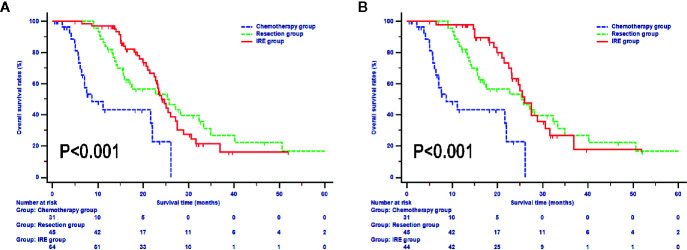
Overall survival analyses stratified by treatments in all included patients **(A)** and patients with neoadjuvant chemotherapy **(B)**.

In the whole study, the median OS during follow-up was 16.9 months (95% CI, 15.4–19.2 months). The median OS of patients in the chemotherapy was 8.7 months (95% CI, 4.4–22.0 months), which was significantly shorter than that of the resection and IRE groups (P < 0.001). Additionally, patients in the resection and IRE groups had similar median OS time [resection group vs. IRE group: 25.3 months (95% CI, 15.9–33.3 months) vs. 24.0 months (95% CI, 22.7–27.5 months), P > 0.050, **Figure 2**]. Survival rates between these two groups were also comparable (resection group vs. IRE group: 1-year OS rate 81.8% vs. 96.8%, 2-year OS rate 53.2% vs. 49.8%, 3-year OS rate 26.7% vs. 16.1%, P > 0.050). Furthermore, the survival of patients who received neoadjuvant chemotherapy in the IRE group was analyzed. The OS of these patients was even more close to that in the resection group (median survival, 26.0 months vs. 25.3 months, P > 0.050, **Figure 2B**).

**Figure 3:**
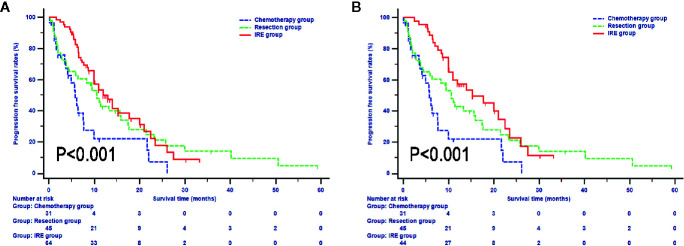
Progression free survival analyses stratified by treatments in all included patients **(A)** and patients with neoadjuvant chemotherapy **(B)**.

In terms of PFS, compared with patients in the resection and IRE groups, those in the chemotherapy group (median survival 5.7 months, 95% CI, 3.8-7.7 months) experienced significantly lower PFS rates (P = 0.009, **Figure 3**). Also, the survival differences of PFS in patients between resection and IRE groups were not significant (P > 0.050). The median PFS for patients in the IRE group was 12.0 months (95% CI, 10.0–17.7 months), with 1-year, 2-year, and 3-year PFS rates of 53.6%, 17.8%, and 8.9%, respectively. Similar median survival rate was observed in patents in the resection group (median PFS 10.6 months, 1-year PFS rate 42.8%, 2-year PFS rate 27.8%, 3-year PFS rate 21.2%). Survival time was further improved in patients after neoadjuvant chemotherapy and the median PFS for patients was 15.2 months (95% CI, 11.0-22.5 months, **Figure 3B**).

### Prognostic Factors for OS and PFS

All clinical and pathological variables were included in the Cox regression analysis. Univariate analysis for OS revealed that gender, tumor size, imaging LN metastasis and treatments were associated with OS. Moreover, multivariate analysis revealed that tumor size and treatments were significant prognostic factor for OS. Compared with chemotherapy, conversional resection (hazard ratio (HR) = 0.274, 95%CI, 0.133–0.564, P < 0.001) and IRE (HR = 0.349, 95%CI, 0.177–0.686, P = 0.002) both predicted better OS (**Table 2**). In terms of PFS, WBC (HR = 2.904, 95%CI, 1.445 – 5.835, P =0.003), tumor size (HR = 2.065, 95%CI, 1.185–3.598, P =0.010), imaging LN metastasis (HR = 1.879, 95%CI, 1.228–2.873, P =0.004) and treatment (HR = 0.446, 95%CI, 0.261–0.761, P =0.003) were associated with PFS. In addition, treatment was also identified by multivariate analysis as a significant prognostic factor. IRE (HR = 0.529, 95%CI, 0.229–0.779, P =0.045) could significantly prolong PFS in LAPC patients, compared with chemotherapy alone (**Table 3**).

**Table 2:** Independent prognostic factors for overall survival (OS).

Characteristics		Univariate analysis			Multivariate analysis		
		HR	95%CI	P	HR	95%	P
Age	≤60 years	Reference		0.827			NI
	>60 years	0.952	0.610–1.484				
Gender	Male	Reference		0.032	Reference		
	Female	1.632	1.042–2.556		1.793	0.984–2.965	0.063
WBC (*10^9^)	≤10	Reference		0.376			NI
	>10	1.461	0.631–3.384				
HGB (g/L)	≤120	Reference		0.701			NI
	>120	1.093	0.694–1.722				
PLT (*10^9^)	≤100	Reference		0.839			NI
	>100	0.946	0.552–1.619				
ALT (U/L)	≤40	Reference		0.377			NI
	>40	0.815	0.518–1.283				
AST (U/L)	≤40	Reference		0.062			NI
	>40	0.638	0.398–1.023				
ALP (U/L)	≤100	Reference		0.376			NI
	>100	0.818	0.524–1.276				
GGT (U/L)	≤45	Reference					NI
	>45	0.783	0.502–1.223	0.282			
ALB (g/L)	≤40	Reference					NI
	>40	0.821	0.494–1.367	0.449			
TBIL (umol/L)	≤20.5	Reference					NI
	>20.5	0.776	0.492–1.224	0.276			
IBIL (umol/L)	≤15	Reference					NI
	>15	0.962	0.555–1.668	0.890			
CRP (ng/L)	≤3	Reference					NI
	>3	0.956	0.612–1.493	0.842			
CEA (ng/mL)	≤5	Reference		0.552			NI
	>5	1.144	0.734–1.785				
CA19-9 (U/ml)	≤35	Reference		0.110			NI
	>35	1.570	0.903–2.728				
Tumor size (cm)	≤2	Reference			Reference		
	2~4	1.274	0.681–2.382	0.449	1.418	0.714–2.817	0.318
	>4	2.663	1.417–5.008	0.002	2.075	0.980–4.395	0.056
Tumor site	Head	Reference					NI
	Body/Tail	1.150	0.821–1.609	0.417			
Treatment	Chemotherapy	Reference			Reference		
	Conversional surgery	0.269	0.141–0.513	<0.001	0.274	0.133–0.564	<0.001
	Chemotherapy + IRE	0.266	0.146–0.485	<0.001	0.349	0.177–0.686	0.002
Chemotherapy type	S-1	Reference					NI
	AG	0.592	0.256–1.369	0.220			
	FOLFIRINOX	1.248	0.717–2.172	0.433			
Imaging LN metastasis	Absence	Reference			Reference		
	Present	1.785	1.123–2.838	0.014	1.045	0.598–1.826	0.878
HBsAg	No	Reference					NI
	Yes	1.311	0.630–2.730	0.469			

OS, overall survival; HR, hazard ratio; CI, confidence interval; NI, not include, other abbreviations as in **Table 1**.

**Table 3:** Independent prognostic factors for progression-free survival (PFS).

Characteristics		Univariate analysis			Multivariate analysis		
		HR	95%CI	P	HR	95%	P
Age	≤60 years	Reference		0.960			NI
	>60 years	1.010	0.674–1.514				
Gender	Male	Reference		0.192			NI
	Female	1.311	0.873–1.968				
WBC (*10^9^)	≤10	Reference		0.003	Reference		
	>10	2.904	1.445–5.835		3.016	0.950–6.274	0.065
HGB (g/L)	≤120	Reference		0.732			NI
	>120	0.930	0.615–1.407				
PLT (*10^9^)	≤100	Reference		0.949			NI
	>100	1.016	0.622–1.659				
ALT (U/L)	≤40	Reference		0.617			NI
	>40	1.109	0.739–1.665				
AST (U/L)	≤40	Reference		0.685			NI
	>40	0.917	0.603–1.395				
ALP (U/L)	≤100	Reference		0.950			NI
	>100	1.013	0.676–1.518				
GGT (U/L)	≤45	Reference					NI
	>45	0.885	0.591–1.326	0.555			
ALB (g/L)	≤40	Reference					NI
	>40	0.857	0.541–1.357	0.510			
TBIL (umol/L)	≤20.5	Reference					NI
	>20.5	0.869	0.575–1.314	0.506			
IBIL (umol/L)	≤15	Reference					NI
	>15	1.130	0.688–1.857	0.629			
CRP (ng/L)	≤3	Reference					NI
	>3	1.141	0.760–1.714	0.524			
CEA (ng/mL)	≤5	Reference		0.645			NI
	>5	1.100	0.734–1.647				
CA19-9 (U/ml)	≤35	Reference		0.060			NI
	>35	1.623	0.979–2.689				
Tumor size	≤2	Reference		0.049	Reference		
	2~4	1.719	1.004–2.945		1.429	0.801–2.550	0.227
0.227	>4	2.065	1.185–3.598	0.010	1.213	0.622–2.366	0.571
Tumor site	Head	Reference					NI
	Body/Tail	1.093	0.799–1.494	0.578			
Treatment	Chemotherapy	Reference			Reference		
	Conversional surgery	0.530	0.303–0.927	0.026	0.562	0.303–1.044	0.068
	Chemotherapy + IRE	0.446	0.261–0.761	0.003	0.529	1.169–0.279	0.042
Chemotherapy type	S-1	Reference					NI
	AG	0.635	0.328–1.227	0.176			
	FOLFIRINOX	0.751	0.452–1.248	0.269			
Imaging LN metastasis	Absence	Reference			Reference		
	Present	1.879	1.228–2.873	0.004	1.502	0.929–2.428	0.097
HBsAg	No	Reference					NI
	Yes	0.708	0.327–1.532	0.380			

PFS, progression free survival, other abbreviations as in **Table 1**.

### Subgroup Survival Analysis in the IRE Group

**Figure 4:**
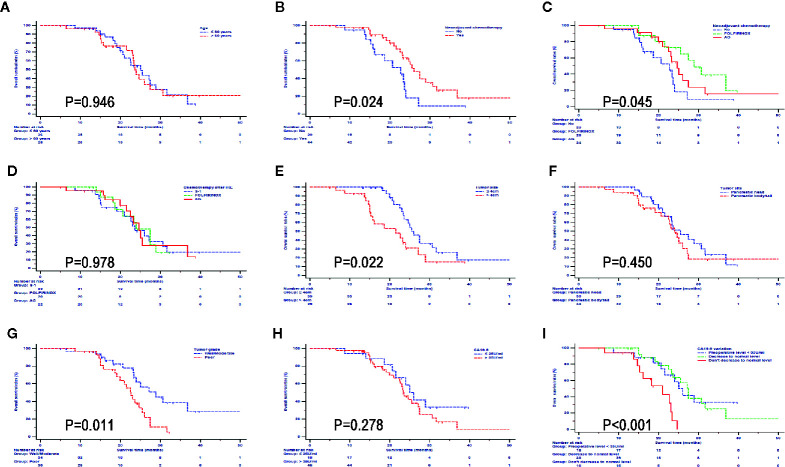
Overall survival analyses stratified by clinical and pathological characteristics in all patients treated with irreversible electroporation (IRE) and chemotherapy. **(A)** age; **(B)** neoadjuvant chemotherapy; **(C)** neoadjuvant chemotherapy regimens; **(D)** Regimens of chemotherapy after IRE; **(E)** Tumor size; **(F)** Tumor site; **(G)** Tumor grade; **(H)** CA19-9; **(I)** CA19-9 variations.

**Figure 5:**
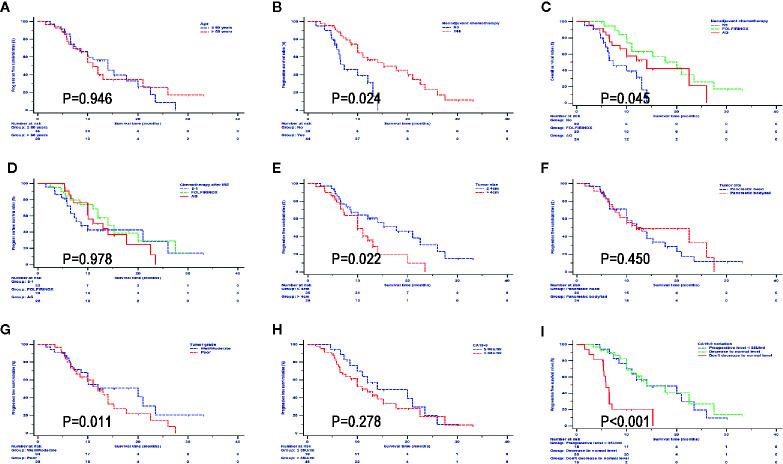
Progression free survival analyses stratified by clinical and pathological characteristics in all patients treated with irreversible electroporation (IRE) and chemotherapy. **(A)** age; **(B)** neoadjuvant chemotherapy; **(C)** neoadjuvant chemotherapy regimens; **(D)** Regimens of chemotherapy after IRE; **(E)** Tumor size; **(F)** Tumor site; **(G)** Tumor grade; **(H)** CA19-9; **(I)** CA19-9 variations.

In order to further investigate the impact from clinical and pathological characteristics in LAPC patients after IRE therapy, survival analyses based on different factors were conducted. It was shown that neoadjuvant chemotherapy, FOLFIRINOX, tumor smaller than 4 cm, and well differentiated tumor predicted better OS (**Figure 4**) and PFS (**Figure 5**). Compared with CA19-9 levels higher than 35 U/ml, patients with lower levels of 35 U/ml had better OS and PFS. In addition, in patients with preoperative CA19-9 levels higher than 35 U/ml, CA19-9 decreased to normal level in 2 months after IRE treatment indicated better survival for LAPC patients after IRE treatment, which was similar to that of patients whose CA19-9 levels were lower than 35 U/ml. Different ages, chemotherapy regimens, tumor sites and raw CA19-9 levels did not have significant impact on survival.

## Discussion

As a malignant tumor, pancreatic cancer has an extremely poor prognosis, which brings great challenge to the treatment. The small proportions of suitable patients for surgery may partly contribute to the poor survival of pancreatic cancer. Chemotherapy is the recommended treatment for PDAC. Recent studies have also consolidated the fundamental role of chemotherapy for its potential role in decreasing metastases in the treatment of this disease. Moreover, it was shown that a large proportion of LAPC patients died of local tumor progression other than distant metastases (11), indicating that on the basis of chemotherapy, strategies to optimize local control may play an increasing role in maximizing therapy in the treatment of LAPC (12).

With the development of neoadjuvant chemotherapy and the advances of surgical techniques, more and more LAPC became resectable and conversional surgery had brought better survival for these patients, compared with conventional chemotherapy (3, 13). In a multicenter study, Philip et al. indicated that chemotherapy combined with surgical resection significantly improved the survival of LAPC patients with a median OS of 18.8 months, compared with those who received chemotherapy only (2). Additionally, Janet et al. reported that LAPC patients who had received chemotherapy with FOLFIRINOX had a median OS of 31.4 months and those who had received additional surgical resection had a median OS of 33.0 months. Obviously, a more effective chemotherapy regimen could further provide better basis for resection and continuously improve survival. However, the conversional rate varied in a great range in different surgical centers from 0% to 43% (3), indicating that the judgement of resectability of LAPC after chemotherapy was influenced by many factors, including chemotherapy regimens, tumor heterogeneity, and surgical technology. Moreover, even the diseases were converted to resectable ones after neoadjuvant chemotherapy, the R0 resection rate of these tumors was only around 78.4%. This may partly because the recommendation of surgical exploration was just made as long as the tumor progression was not detected after neoadjuvant chemotherapy (10). Additionally, it should be noticed that the involvement of major vessels was not significantly improved in such a great part of LAPC after neoadjuvant chemotherapy. Extended surgery including vascular resection is often necessary in order to achieve tumor-free resection margins. A relatively high postoperative complications of extended surgery compared with standard pancreatectomy would also decrease survival to some degrees (14). Considering the tumor biology and the response to neoadjuvant chemotherapy, usually surgical resection was performed in patients whose CA19-9 levels decreased by more than a half after neoadjuvant chemotherapy (10). Therefore, although extended surgery may provide additional survival benefit for selected patients, it should be considered carefully as the potential complications may be associated with inferior survival.

Compared with conversional resection for LAPC, IRE may be a much less invasive method which aims at locally destroying tumor. The safety and effectiveness of IRE have been reported by many studies (15, 16). Moreover, it was shown that IRE combined with chemotherapy elevated survival significantly compared with chemotherapy alone (8, 9). In the study conducted by Martin et al., LAPC patients had a median OS of 24.9 months and PFS of 12.4 months after IRE and chemotherapy treatment (5). Similar survival results were also observed in this study, with the median OS and PFS of 24.0 and 12.0 months for LAPC patients after IRE treatment. Additionally, for LAPC patients who had received neoadjuvant chemotherapy and IRE, the survival was even higher with a median OS of 26.0 months and a median PFS of 15.2 months. Compared with receiving conversional resection, patients receiving IRE treatment after chemotherapy had even higher survival rates, although the survival differences were not significant. Also, the survival results of IRE group in this study was comparable to that of LAPC patients after conversional resection which was reported in a pooled patient-level study (3). Additionally, as the most powerful chemotherapy regimens for pancreatic cancer, FOLFIRINOX only or FOLFIRINOX combined with conversional resection was reported to elevate the survival of LAPC patients to 31.4 and 33.0 months, respectively (13), illustrating that the neoadjuvant chemotherapy was the basis of the combination treatment. Neoadjuvant chemotherapy combined with conversional resection or IRE could also further improve the survival of LAPC patients. In the present study, only a small proportion of patients have received chemotherapy with full doses of FOLFIRINOX. This might partly explain the reason for the inferior survival compared with that of patients after chemotherapy with full doses of FOLFIRINOX. Therefore, it is believed that FOLFIRINOX combined with IRE would further improve the survival of LAPC patients.

As a novel therapy for LAPC, IRE combined with chemotherapy provided varied survival benefit for patients with different characteristics. Subgroup survival analyses illustrated that neoadjuvant chemotherapy, chemotherapy with FOLFIRINOX, tumor smaller than 4 cm, well differentiated tumor and CA19-9 decreased to normal level in two months after IRE treatment predicted better survival. Therefore, it implied that the evaluation of tumor characteristics before IRE was important. The neoadjuvant chemotherapy, which aimed to eliminate some potential micrometastases, was extremely important for the cancer control. In terms of tumor sizes, patients whose tumors smaller than 4 cm had better survival than those with larger tumor sizes, which was similar with results from other studies (5, 17). The incomplete ablation was more common in tumors with large tumor sizes, which might contribute to the inferior survival of LAPC patients after IRE treatment. Similar with previous study (18), our study also indicated that the changes of CA19-9 levels were an important reflection of tumor response of IRE. High levels of CA19-9 predicted poor survival in LAPC patients after IRE treatment. Additionally, in patients with preoperative CA19-9 levels higher than 35 U/ml, those whose CA19-9 levels decreased to normal level had similar survival rates to those whose preoperative CA19-9 levels were normal. Since a small change in CA19-9 has no clinical implications, 50% of the initial CA19-9 levels was regarded as the threshold of CA19-9 changes in this study. Patients with a decrease by more than a half in CA19-9 level had a better prognosis. This implies that the use of changes of CA19-9 level after IRE treatment as a prognostic marker. Additionally, this might indicate which patients are suitable for an aggressive approach with chemotherapy post IRE with the intent of reaching a setting for radical treatment. Compared with chemotherapy, the conversional resection and IRE combined with chemotherapy significantly prolonged survival time in LAPC patients. In addition, compared with conversional resection, the similar survival results and much less invasive nature makes IRE a suitable method for the treatment of LAPC after conventional chemotherapy.

There were several limitations. First, there was potential selection bias in the retrospective trial. Second, the numbers of included patients were not large enough for a firm conclusion. Further prospective studies based on large cohorts are needed to consolidate the conclusion of this study. Third, some indices which could reflect the safety and effectiveness of IRE or conversional resection, such as complications, were not included in this study. Further evaluation of complications would provide a more comprehensive overview of these two therapies.

In conclusion, IRE combined with chemotherapy and conversional resection shared similar survival rates in LAPC patients, which was significantly higher than those of patients treated by chemotherapy alone. The less invasive nature makes IRE a considerable treatment for LAPC. A randomized clinical trial comparing the efficacy of these three methods is therefore warranted.

## Data Availability Statement

The raw data supporting the conclusions of this article will be made available by the authors, without undue reservation.

## Ethics Statement

The studies involving human participants were reviewed and approved by Institutional Review Board of Sun Yat-sen University Cancer Center. The patients/participants provided their written informed consent to participate in this study.

## Author Contributions

SL was responsible for conception, design and quality control of this study. CH, SS, XH, and YZ performed the study selection, data extraction, statistical analyses, and was major contributors in writing the manuscript. CH, SS, and XH participated in studies selection and statistical analyses. CH, SS, XH, YZ, and XL contributed in classification criteria discussion. CH, SS, and XH contributed to the writing of manuscript. SL reviewed and edited the manuscript respectively. All authors contributed to the article and approved the submitted version.

## Funding

This work was supported by grants from the National Natural Science Funds (No.81972299, No. 81672390) and the National Key Research and Development Plan (No.2017YFC0910002).

## Conflict of Interest

The authors declare that the research was conducted in the absence of any commercial or financial relationships that could be construed as a potential conflict of interest.
